# 

**Published:** 1883-12

**Authors:** 


WHOLE NUMBER, CCLIX.

DECEMBER, 1883.

NUMBER 5, VOLUME XXIII.

### THE BUFFALO Medical and Surgical Journal.

LIBRARY INDEXED S.G.O.

EDITORS:

THOS. LOTHROP, M. D., A. R. DAVIDSON, M. D.,

P. W. VAN PEYMA, M. D.

PUBLISHED BY THE MEDICAL JOURNAL ASSOCIATION.

No. 5 WEST CHIPPEWA ST.

Entered at the Post-Office at Buffalo, N. Y., as Second-class Mail Matter.

